# Diagnostic puzzles in primary amenorrhea: a report of 3 cases

**DOI:** 10.1210/jcemcr/luag197

**Published:** 2026-07-28

**Authors:** Sapna Nayak, Sonali Verma, Ankur Mandelia, Preeti Dabadghao, Margaret Zacharin

**Affiliations:** Department of Endocrinology, Sanjay Gandhi Postgraduate Institute of Medical Sciences, Lucknow, Uttar Pradesh 226014, India; Division of Pediatric Endocrinology, Department of Pediatrics, Christian Medical College, Vellore, Tamil Nadu 632004, India; Department of Endocrinology, Sanjay Gandhi Postgraduate Institute of Medical Sciences, Lucknow, Uttar Pradesh 226014, India; Pediatric Endocrinology, Aria Healthcare, Deoghar, Jharkhand 814112, India; Department of Pediatric Surgery, Sanjay Gandhi Postgraduate Institute of Medical Sciences, Lucknow, Uttar Pradesh 226014, India; Department of Endocrinology, Sanjay Gandhi Postgraduate Institute of Medical Sciences, Lucknow, Uttar Pradesh 226014, India; Department of Pediatrics, University of Melbourne, Parkville, Victoria 3052, Australia; Department of Endocrinology, Royal Children's Hospital, Murdoch Children′s Research Institute, Parkville, Victoria 3052, Australia

**Keywords:** Mullerian agenesis, MRKH, ectopic ovaries, MURCS, primary amenorrhea, lipomeningomyelocele

## Abstract

Primary amenorrhea is a common referral indication in pediatric and adolescent endocrinology and can occur due to hypothalamic-pituitary disorders, uterovaginal anatomical anomalies, or gonadal dysfunction. We describe 3 adolescents presenting with primary amenorrhea and otherwise appropriate pubertal development, due to Mayer-Rokitansky-Kuster-Hauser syndrome, each illustrating a distinct diagnostic challenge. The patient in case 1, who also had concerns of short stature, had elevated gonadotropin levels, suggesting a possibility of primary ovarian insufficiency. However, a normal estradiol level and repeat gonadotropin testing clarified the underlying hormonal physiology. The patient in case 2 presented with bilateral inguinal swellings and primary amenorrhea mimicking androgen insensitivity, but imaging demonstrated ectopic ovaries with absent Müllerian structures. The patient in case 3 had primary amenorrhea and a large lumbosacral mass with neurological symptoms, due to a lipomeningomyelocele associated with Müllerian agenesis. A structured evaluation incorporating repeat hormonal assessment and detailed imaging clarified the diagnosis in all cases. This report highlights the common diagnostic pitfalls during evaluation for primary amenorrhea and emphasizes the importance of reviewing the biochemical data in the context of clinical and imaging findings. In addition, a retrospective review of our cohort of adolescent girls with similar presentations is elaborated to highlight the range of associated findings.

## Introduction

Primary amenorrhea is the absence of menarche by the age of 15 years with otherwise normal pubertal development or within 3 years after thelarche [1]. The wide etiological spectrum includes hypothalamic or pituitary dysfunction, systemic disorders, and gonadal dysfunction, all of which disturb the hormonal physiology. The hypothalamic-pituitary-gonadal (ovarian) (HPG) axis in females drives the menstrual (endometrial) cycle. Anatomical causes, such as Müllerian agenesis (Mayer-Rokitansky-Kuster-Hauser [MRKH] syndrome) or genital tract outflow obstruction, have intact ovarian function and hormonal levels [2]. MRKH syndrome either occurs in isolation (type I) or with extragenital anomalies (type II), which include Müllerian duct aplasia, renal aplasia, and cervicosomatic dysplasia (MURCS) association. Because the HPG axis remains functional with cyclical variation in these girls, caution must be exercised when interpreting laboratory results to avoid misdiagnosis.

## Case presentation

### Case 1

An 18-year-old female presented with concerns of short stature, primary amenorrhea, and cyclical lower abdominal pain. Spontaneous thelarche and pubarche occurred at 12 to 13 years with no history of virilization, galactorrhea, or previous exposure to any exogenous hormones. On physical examination, her height was 151 cm (−1.2 SD score) with a sexual maturity rate of Tanner stage V. She had an increased carrying angle but no other overt skeletal deformities. Genital examination revealed no bluish bulging membrane, but a blind vaginal pouch was suspected with normal estrogenization of the external genitalia and vaginal mucosa.

### Case 2

A 16.5-year-old girl presented with primary amenorrhea and a congenital right inguinal swelling that had enlarged and become painful over the last 4 months. Examination revealed normal stature, blood pressure, breast and pubic hair development (Tanner stage IV), no clitoromegaly, and a blind vaginal pouch. Firm, tender, palpable inguinal masses were identified bilaterally (approximately 3 × 4 cm on the right and 2 × 2 cm on the left). Bilateral inguinal swellings in a girl with primary amenorrhea and normal breast development raised the possibility of complete androgen insensitivity syndrome (CAIS).

### Case 3

A 17-year-old female presented with primary amenorrhea and a history of urinary incontinence, untreated. Recently, she developed numbness and distal muscle weakness in her lower limbs. Examination revealed hypotonia, absent deep tendon reflexes in both lower limbs, and absent plantar response bilaterally. A tender, skin-covered lumbosacral mass was also palpable.

## Diagnostic assessment

### Case 1

**Figure 1:**
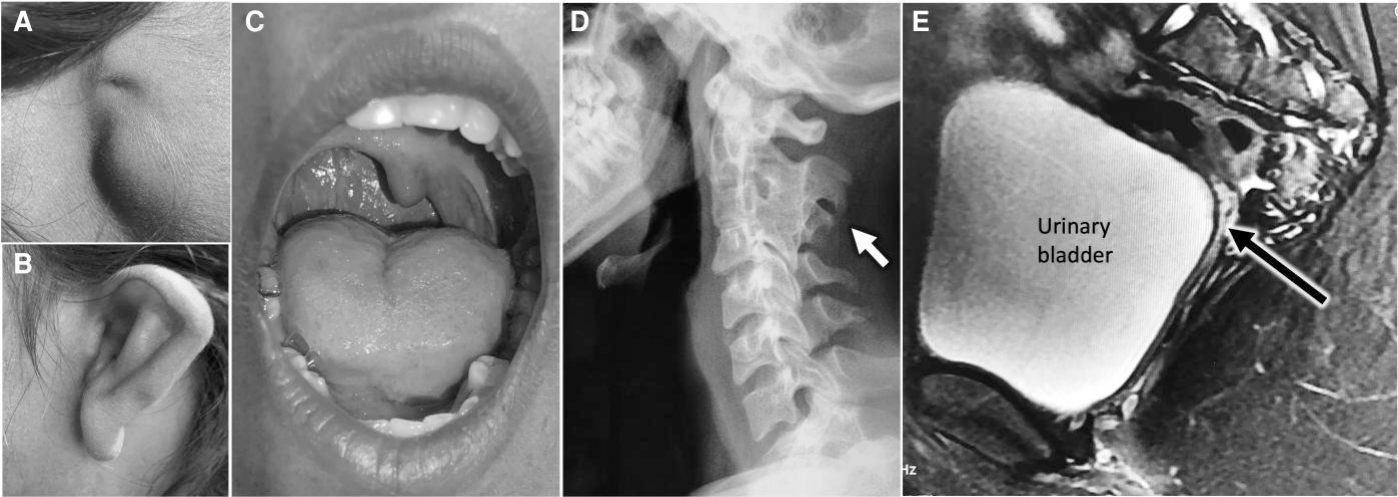
Clinical and imaging findings in case 1: (A) right anotia; (B) left abnormally shaped pinna; (C) asymmetry of the soft palate; (D) lateral radiograph of cervical spine showing fusion of cervical vertebrae C2, C3, and C4 (white arrow); and (E) sagittal section of magnetic resonance imaging pelvis showing rudimentary uterus (black arrow).

Initial blood investigations showed elevated levels of luteinizing hormone (LH) (33.4 mIU/mL [33.4 IU/L]; reference range [RR], 0.9-14.7 mIU/mL [0.9-14.7 IU/L]) and follicle-stimulating hormone (FSH) (21.5 mIU/mL [21.5 IU/L]; RR, 0.6-10.8 mIU/mL [0.6-10.8 IU/L]), raising suspicion of hypergonadotropic hypogonadism, likely related to Turner syndrome, particularly as she was a little short. However, she had clearly achieved her mid-parental height expectation (155 cm). The LH-predominance and a modestly elevated FSH, with a normal serum estradiol level (235 pg/mL [862 pmol/L]; RR, 26-355 pg/mL [95-1303 pmol/L]) argued against the possibility of primary ovarian insufficiency. A more careful systemic examination revealed associated congenital anomalies, including facial asymmetry, right anotia, and small malformed left pinna, consistent with a history of longstanding hearing difficulty, which she had previously not disclosed. Oral examination demonstrated asymmetry of the soft palate without clefting (Fig. 1A-1C).

Repeat hormonal assessment performed 6 weeks later demonstrated normal luteal phase values (LH, 0.35 mIU/mL [0.35 IU/L]; FSH, 3.3 mIU/mL [3.3 IU/L]; estradiol, 28 pg/mL [104 pmol/L]), confirming intact HPG axis function and suggesting that the high initial gonadotropin levels were due to a midcycle hormonal surge. Having established normal functioning of the HPG axis, the differential diagnosis now narrowed to anatomical defects of the genital tract. Pelvic ultrasonography (USG) followed by magnetic resonance imaging (MRI) showed a rudimentary uterus, establishing the diagnosis of MRKH syndrome. Both kidneys were normal. Further, a radiograph of the spine showed fusion of cervical vertebrae C2-C3-C4 (Fig. 1D and 1E).

### Case 2

**Figure 2:**
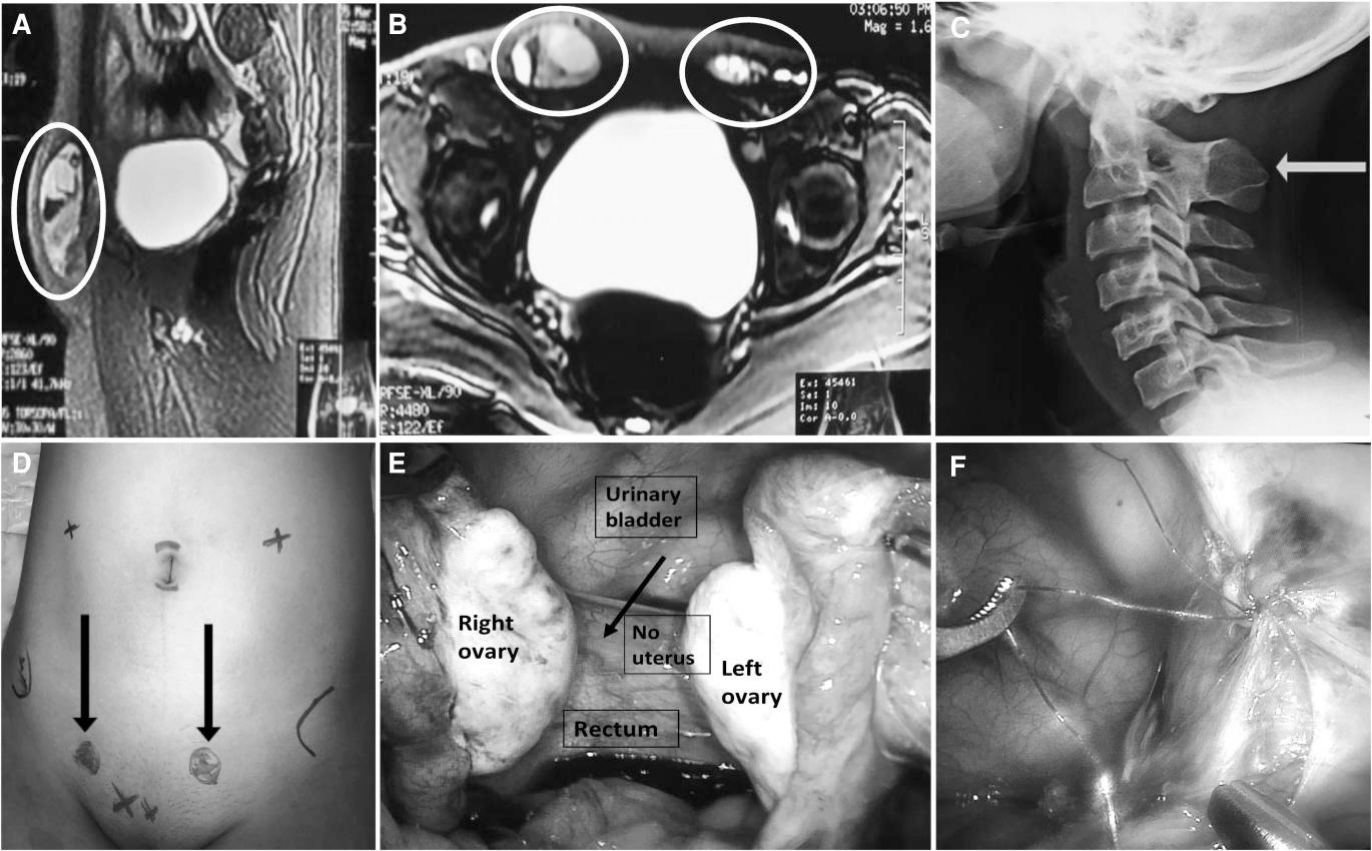
Imaging and intraoperative findings in case 2 with Müllerian agenesis and bilateral ovarian ectopia: (A) sagittal and (B) transverse sections of magnetic resonance imaging pelvis showing absence of uterus with ectopic edematous ovaries located in the parietal wall (white ovals); (C) lateral radiograph of cervical spine showing fusion of cervical vertebrae C2 and C3 (white arrow); (D) intraoperative image showing marking of the positions of ectopic ovaries (black arrows) and laparoscopic ports (cross marks); (E) both ovaries after reduction into the abdomen; (F) inguinal canal repair.

Initial USG identified the inguinal swellings as ectopic ovaries located at the superficial inguinal rings, each with follicles and an echogenic central stroma. The uterus, cervix, and the left kidney were absent, and the right kidney was enlarged (11.5 cm). MRI confirmed these findings and demonstrated massive edema in the right ovary (6.9 cm × 3.6 cm). Normal gonadotropin levels indicated preserved ovarian function (Table 1). She was diagnosed with MRKH type II with bilateral ovarian ectopia in the inguinal canals, a rare presentation. Radiograph of the spine revealed fusion of the C2-C3 vertebrae (Fig. 2A-2C).

**Table 1:** The clinical features and hormonal profile in our cohort of girls with Mayer-Rokitansky-Kuster-Hauser syndrome

Case no.	Presenting age, years	Associated features	Hormonal values
1	18	Facial features, right anotia, left malformed pinna, asymmetric soft palate Fusion of cervical vertebrae C2-C3-C4Fig. 1)	LH: 33.4 mIU/mL (33.4 IU/L) FSH: 21.5 mIU/mL (21.5 IU/L) E2: 235 pg/mL (862 pmol/L) Repeat testing*^a^*: LH: 0.35 mIU/mL (0.35 IU/L) FSH: 3.3 mIU/mL (3.3 IU/L) E2: 28 pg/mL (104 pmol/L)
2	16.5	Ectopic ovaries (Fig. 2) C2-C3 fusion Solitary right kidney	LH: 9.8 mIU/mL (9.8 IU/L) FSH: 6.6 mIU/mL (6.6 IU/L) E2: <20 pg/mL (<73.6 pmol/L) T: 31.7 ng/dL (1.1 nmol/L)
3	17	Ectopic left kidney (pelvic) Lumbar scoliosis, spina bifida with lipomeningomyelocele (Fig. 3) Partial fusion of dorsal vertebrae D3-D4	LH: 2.8 mIU/mL (2.8 IU/L) FSH: 3.9 mIU/mL (3.9 IU/L) E2: 90 pg/mL (332 pmol/L) PRL: 404 mIU/L (19 µg/L)
4	12.5	Crossed-fused renal ectopiaFig. 4)C2-C3 fusion	LH: 2.7 mIU/mL (2.7 IU/L) FSH: 2.6 mIU/mL (2.6 IU/L) E2: 63 pg/mL (232 pmol/L)
5	15	Solitary right kidney Left ovarian agenesis (nonvisualized ovary on MRI)	LH: 3.7 mIU/mL (3.7 IU/L) FSH: 4.5 mIU/mL (4.5 IU/L) E2: NA PRL: 372 mIU/L (17.5 µg/L)
6	17	Solitary right kidney	LH: 7.0 mIU/mL (7.0 IU/L) FSH: 3.3 mIU/mL (3.3 IU/L) E2: NA
7	16.5	Tall stature Height: 171 cm, +2.1 SDS, MPH: 153 cm	LH: 1.6 mIU/mL (1.6 IU/L) FSH: 4.9 mIU/mL (4.9 IU/L) E2: 35 pg/mL (130 pmol/L) T: 14 ng/dL (0.5 nmol/L) IGF-1: 373 ng/mL (48.7 nmol/L)
8	14.5	Short stature Height: 142 cm, −2.07 SDS, MPH: 152 cm	LH: 9.7 mIU/mL (9.7 IU/L) FSH: 6.2 mIU/mL (6.2 IU/L) E2: NA PRL: 351 mIU/L (16.5 µg/L)
9	16	None	LH: 6.8 mIU/mL (6.8 IU/L) FSH: 2.2 mIU/mL (2.2 IU/L) E2: 64 pg/mL (236 pmol/L) PRL: 212 mIU/L (10 µg/L)
10	25	None	LH: 9.0 mIU/mL (9.0 IU/L) FSH: 4.2 mIU/mL (4.2 IU/L) E2: 119 pg/mL (437 pmol/L)

Six girls (cases 1-6) were classified as having the Mayer-Rokitansky-Kuster-Hauser (MRKH) type II in the presence of additional findings, while 4 (cases 7-10) had isolated Müllerian agenesis (MRKH type I). MRI of the pelvis confirmed the diagnosis with findings of an absent uterus in 7 girls and a rudimentary uterus in the remainder. Renal anomalies were observed in 5/10 girls (solitary kidney in 3, ectopic kidney in 1, and crossed-fused ectopia in 1 [Fig. 4]). Occult spinal anomalies were found on radiographs in 3 girls (cases 1, 2, and 4). No hormonal abnormalities causing tall stature were found in case 7. Normal reference ranges for pubertal females: E2, 26-355 pg/mL (95-1303 pmol/L); FSH, 0.6-10.8 mIU/mL (0.6-10.8 IU/L); IGF-1, 208-620 ng/mL (27.2-81 nmol/L); LH, 0.9-14.7 mIU/mL (0.9-14.7 IU/L); PRL, <532 mIU/L (<25 µg/L); and T, <40.3 ng/dL (<1.4 nmol/L).

Abbreviations: E2, estradiol; FSH, follicle-stimulating hormone; IGF-1, insulin-like growth factor-1; LH, luteinizing hormone; MPH, midparental height; MRI, magnetic resonance imaging; NA, not available; PRL, prolactin; SDS, SD score; T, testosterone.

^*a*^In view of perplexing results, the hormonal tests were repeated after a 6-week duration.

### Case 3

**Figure 3:**
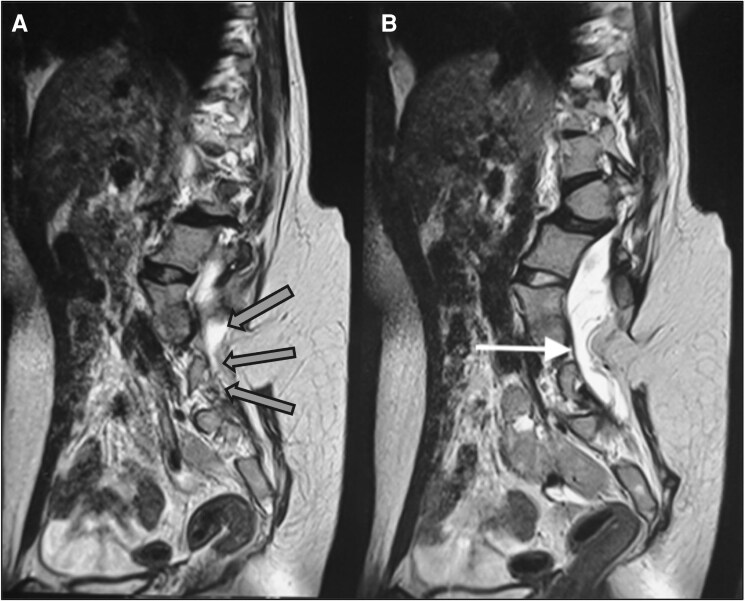
Sagittal section of magnetic resonance imaging spine in case 3 showing (A) defect in the posterior element of L5 vertebra (grey arrows) with (B) fat-containing lesion of size 2.0 × 4.0 × 3.5 cm (white arrow) in the posterior paraspinal soft tissue at the lumbosacral region communicating with the thecal sac, suggestive of spina bifida with lipomeningomyelocele.

MRI of the spine showed lumbar spina bifida with lipomeningomyelocele (Fig. 3), partial fusion of the dorsal vertebrae and multiple vertebral hemangiomas. Findings of Müllerian agenesis on pelvic MRI, normal gonadotropin levels (Table 1), and vertebral defects were consistent with MRKH type II.

**Figure 4:**
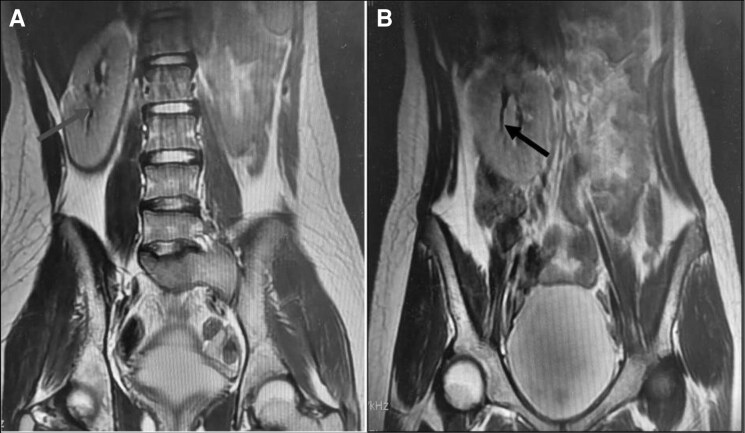
Coronal section of magnetic resonance image of the abdomen in case 4 described in Table 1, showing crossed-fused renal ectopia on the right side with (A) one kidney with the pelvis oriented outward (grey arrow) and (B) the second kidney with pelvis oriented inward (black arrow) and fused with the other kidney.

To place these cases in a broader perspective, a retrospective review of the clinical records of our Pediatric Endocrine Clinic over 3 years (between April 2019 and March 2022) was conducted, and 7 additional girls with MRKH syndrome were identified. All patients, including those noted herein, presented with primary amenorrhea at a median age of 16.2 years (range, 12.5-25 years) and had well-developed secondary sexual characteristics. The karyotype was 46,XX in all cases. Their hormonal parameters, MRI findings, and associated anomalies are depicted in Table 1. MRI demonstrated crossed-fused renal ectopia in 1 patient (Fig. 4).

## Treatment

No hormonal treatment was initiated in any of the patients, as ovarian function was intact in all. The families and patients were appropriately counseled regarding the diagnosis, reproductive options, need for long-term follow-up, and creation of a neovagina when age-appropriate.

In case 2, laparoscopic reduction of the ectopic edematous ovaries with bilateral inguinal herniorrhaphy was undertaken (Fig. 2D-2F). The absence of Müllerian structures was confirmed by laparoscopy. Gonadal biopsies showed normal ovarian stroma with primary follicles.

Surgical excision of the lipoma and repair of the defect were planned for case 3 but deferred at the family's request at the time of reporting.

## Outcome and follow-up

All patients remained clinically stable on follow-up. Multidisciplinary referral was arranged for those with renal and skeletal associations.

## Discussion

Among the varied etiologies of primary amenorrhea, anatomical causes are likely in the presence of spontaneous pubertal development and normal gonadotropin levels. Müllerian aplasia or MRKH syndrome is characterized by an aplastic/hypoplastic uterus, absent cervix, and upper vagina in a 46,XX female [3]. MRI of the pelvis is the noninvasive modality of choice for diagnosis, with sensitivity and specificity of up to 100% [4]. Our cases represent unusual presentations of MRKH syndrome, highlighting diagnostic pitfalls that can lead to misdiagnosis.

The markedly elevated gonadotropin levels in case 1 represent a potential diagnostic trap for the unwary. At first glance, such values may be misinterpreted as ovarian dysfunction prompting a divergent diagnostic and management pathway. However, understanding normal ovarian physiology, including the cyclicity of gonadotropins, estrogen, progesterone, and the midcycle preovulatory LH surge, clarifies the mechanism underlying these findings [5]. The higher LH than FSH seen in our case is not typical of primary ovarian insufficiency and immediately suggested a midcycle surge, further confirmed by the high estradiol value and the second postovulatory sample showing normalization of parameters. Since girls with MRKH syndrome do not menstruate, the timing of the ovarian cycle cannot be clinically determined when interpreting hormone levels. Therefore, realizing the pattern of gonadotropin rise, a simultaneous estradiol measurement, and a timed hormonal reassessment when required is crucial and, in our case, confirmed that the elevated gonadotropins reflected normal midcycle physiology. In addition, the presence of facial and auricular anomalies, a rare association with Müllerian agenesis, suggested an underlying anatomical etiology [6].

Although ovarian function is typically normal in MRKH syndrome, large imaging studies have reported ectopic ovaries in approximately one-third of patients, with the majority being located within the pelvis [7]. Ovarian descent into the inguinal canal, presenting as visible or palpable inguinal swellings, has rarely been described [8, 9]. In females with primary amenorrhea and normal breast development, inguinal swellings raise concern for CAIS, typically associated with absent or extremely sparse pubic hair. However, girls with MRKH and inguinal ectopic ovaries presenting in the early stages of puberty may exhibit less pubic hair, potentially mimicking CAIS. In such cases, pelvic USG is valuable in characterizing inguinal structures and differentiating testes (in CAIS) from ectopic ovaries (in MRKH). Hormonal evaluation and karyotyping further assist in cases where imaging is inconclusive. Though ovaries and the uterus develop independently, ovarian descent is guided by the gubernaculum, which anchors the uterus to the inferior ovarian pole and later forms the utero-ovarian and round ligaments. Abnormalities in this pathway resulting in ovarian maldescent occur with greater frequency in girls with Müllerian agenesis than in those with normal uterine anatomy. Thus, when not found in their usual position on MRI, the ovaries should be looked for in ectopic locations. Preservation of the ovaries is generally recommended in such cases. Surgical repositioning of the ovaries into the pelvis, as in our case, reduces the risk of trauma and vascular compromise.

Extragenital malformations reported in nearly half of the individuals with Müllerian agenesis frequently involve the renal and skeletal systems. Spinal anomalies can be occult, such as vertebral fusion and spina bifida occulta, or obvious, such as scoliosis [10-12]. While spinal anomalies are well documented, lipomeningomyelocele, a form of closed spinal dysraphism involving a fatty mass and neural elements, seen in case 3, has not been previously described with MRKH syndrome. The high prevalence of coexisting genitourinary anomalies reflects their shared mesodermal origin and underscores the need to evaluate both systems when either is abnormal.

Genetic alterations in the short-stature homeobox (*SHOX)* gene (duplications) have been reported in cases of both MRKH syndrome and nonfamilial tall stature. Although this represents a plausible mechanism for the combination of Müllerian agenesis and tall stature in 1 of our additional cases (Table 1), this could not be ascertained due to a lack of genetic testing in our cohort and no previously documented associations in the literature [13, 14].

Ovarian agenesis, seen in patient 5 of our cohort, is an extremely uncommon finding described in girls with Müllerian aplasia and a 46,XX karyotype, further illustrating the developmental diversity within this syndrome [15, 16].

A multidisciplinary approach with early psychological counseling for patients and their families is essential. Creation of a neovagina with nonsurgical dilation or, in selected cases, by surgery should be offered in an age-appropriate manner. Options for childbearing, including gestational surrogacy with the patient's own oocytes (as ovarian function remains intact), adoption, and emerging approaches like uterine transplantation, may be discussed within an ethical framework.

## Learning points

- Understanding the ovarian cycle and physiological hormonal fluctuations is essential when interpreting endocrine profiles in females with primary amenorrhea. LH-predominant gonadotropin elevation should prompt consideration of a midcycle surge, before labeling as ovarian insufficiency.
- Lipomeningomyelocele associated with Müllerian agenesis expands the phenotypic spectrum of MRKH syndrome.
- Ovarian anomalies, such as ectopic ovaries or ovarian agenesis, can coexist with Müllerian anomalies alongside renal and skeletal anomalies.

## Contributors

All authors made individual contributions to authorship. All authors were involved in the diagnosis and management of patients. S.N., S.V., and M.Z. were involved in the conceptualization, data collection, drafting, and revision of the manuscript. A.M. was involved in the surgical management of the case involved. P.D. was involved in the conceptualization and revision of the manuscript. All authors reviewed and approved the final draft.

## Data Availability

Data sharing is not applicable to this article as no data sets were generated or analyzed for this case report.
